# High Dengue Burden and Circulation of 4 Virus Serotypes among Children with Undifferentiated Fever, Kenya, 2014–2017

**DOI:** 10.3201/eid2611.200960

**Published:** 2020-11

**Authors:** Melisa M. Shah, Bryson A. Ndenga, Francis M. Mutuku, David M. Vu, Elysse N. Grossi-Soyster, Victoria Okuta, Charles O. Ronga, Philip K. Chebii, Priscilla Maina, Zainab Jembe, Carren M. Bosire, Jael S. Amugongo, Malaya K. Sahoo, ChunHong Huang, Jenna Weber, Sean V. Edgerton, Jimmy Hortion, Shannon N. Bennett, Benjamin A. Pinsky, A. Desiree LaBeaud

**Affiliations:** Stanford University School of Medicine, Stanford, California, USA (M.M. Shah, D.M. Vu, E.N. Grossi-Soyster, M.K. Sahoo, C. Huang, J. Weber, J. Hortion, B.A. Pinsky, A.D. LaBeaud);; Kenya Medical Research Institute, Kisumu, Kenya (B.A. Ndenga, V. Okuta, C.O. Ronga);; Technical University of Mombasa, Mombasa, Kenya (F.M. Mutuku, C.M. Bosire, J.S. Amugongo);; Msambweni County Referral Hospital, Msambweni, Kenya (P.K. Chebii, P. Maina);; Diani Health Center, Ukunda, Kenya (Z. Jembe);; California Academy of Sciences, San Francisco, California, USA (S.V. Edgerton, S.N. Bennett);; École Normale Supérieure de Lyon, Lyon, France (J. Hortion)

**Keywords:** dengue, dengue virus, viruses, arboviruses, serotypes, disease burden, circulation, children, undifferentiated fever, zoonoses, Kenya

## Abstract

Little is known about the extent and serotypes of dengue viruses circulating in Africa. We evaluated the presence of dengue viremia during 4 years of surveillance (2014–2017) among children with febrile illness in Kenya. Acutely ill febrile children were recruited from 4 clinical sites in western and coastal Kenya, and 1,022 participant samples were tested by using a highly sensitive real-time reverse transcription PCR. A complete case analysis with genomic sequencing and phylogenetic analyses was conducted to characterize the presence of dengue viremia among participants during 2014–2017. Dengue viremia was detected in 41.9% (361/862) of outpatient children who had undifferentiated febrile illness in Kenya. Of children with confirmed dengue viremia, 51.5% (150/291) had malaria parasitemia. All 4 dengue virus serotypes were detected, and phylogenetic analyses showed several viruses from novel lineages. Our results suggests high levels of dengue virus infection among children with undifferentiated febrile illness in Kenya.

Dengue virus (DENV) is a reemerging arbovirus with an expansive worldwide range (*1*). Recent modeling studies suggest wider dengue circulation in Africa than previously recognized (*2*). In addition, both major dengue vectors (*Aedes aegypti* and *Ae. albopictus* mosquitoes) are present in Africa (*3*,*4*). The true burden of dengue is likely underestimated because most infections are never accounted for among patients who have self-limited disease or are misdiagnosed as malaria (*2*).

Dengue viremia among returning travelers from Africa and scattered reports from individual countries suggest ongoing dengue circulation in Africa (*2*). Each DENV serotype (DENV-1, DENV-2, DENV-3, and DENV-4) is structured into major lineages referred to as genotypes which have been defined (*5*–*7*). Relatively few DENV sequences are available from Africa (Figure 1), and DENV-2 is the predominant serotype in Africa to date (*2*).

**Figure 1:**
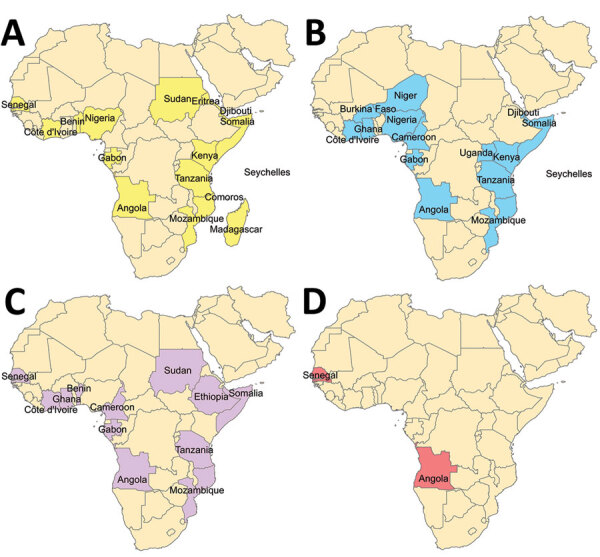
Countries in Africa (indicated by colors) for which dengue virus (DENV) sequences isolated from humans are available in GenBank. A) DENV-1, B) DENV-2, C) DENV-3, D) DENV-4. Further details on search strategy used for this map are available in Appendix Table.

Isolation of DENV-1 and DENV-2 in Africa was made in Nigeria during 1968 (*8*). The first DENV-3 strains isolated from humans from Africa were reported from Mozambique during 1984 (*9*). Only 2 human DENV-4 sequences are publicly available from Africa, 1 from Senegal during 1986 and 1 from Angola during 2014 (*10*).

A laboratory-confirmed outbreak of dengue occurred in coastal Kenya during 1982, followed by a large dengue outbreak in northeastern Kenya during 2011 (*11*,*12*). Circulation of DENV-1, DENV-2, and DENV-3 was reported in northern and coastal Kenya during 2011–2014 (*13*). 

Given the mounting evidence for dengue in Africa, we evaluated the presence of dengue viremia during 4 years of surveillance (2014–2017) among children with febrile illness in coastal and western Kenya by using a highly-sensitive, multiplexed, real-time reverse transcription PCR (rRT-PCR) and genomic sequencing. We report the clinical manifestations, associated factors, serotypes, and phylogenetic relationships.

## Methods

### Study Sites and Enrollment

**Figure 2:**
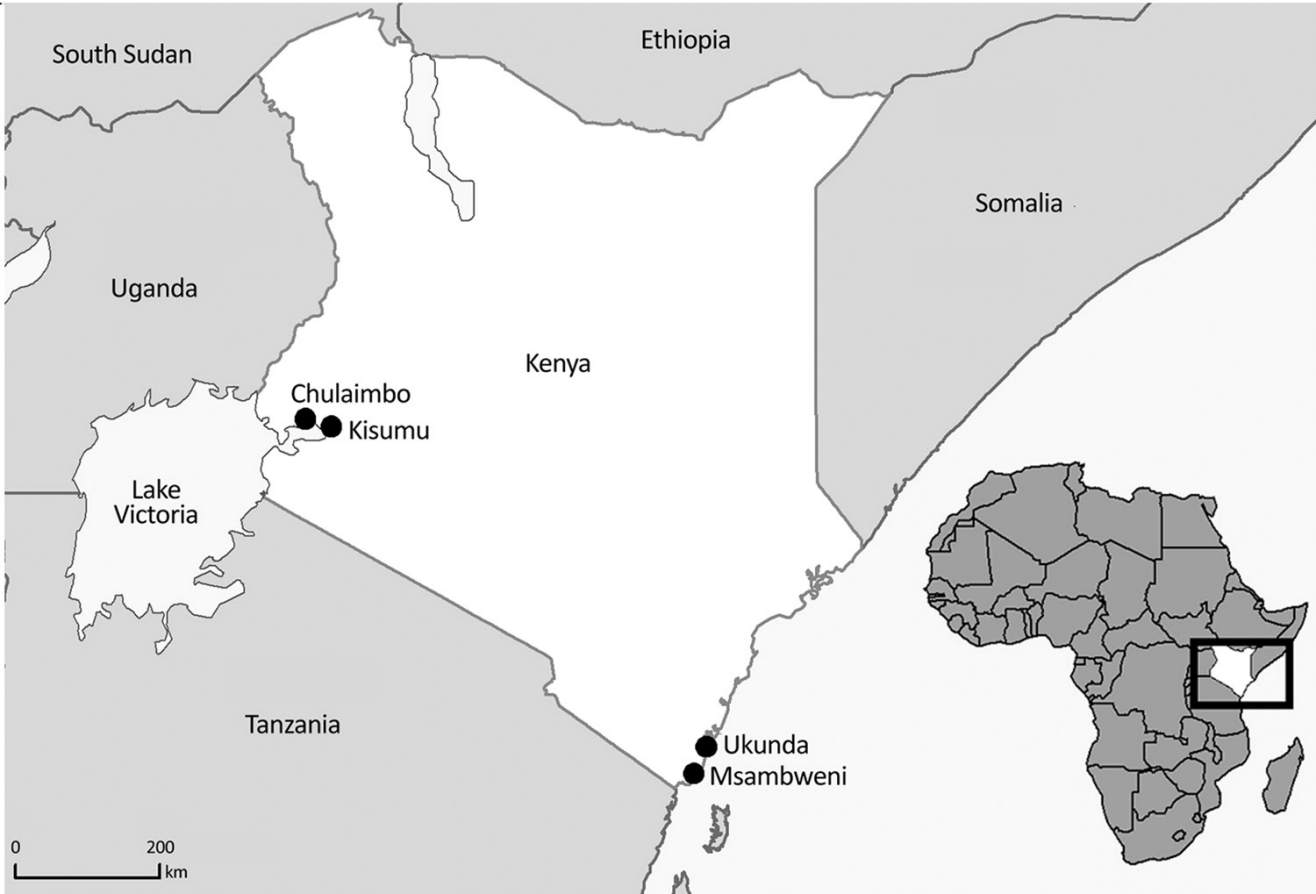
Study recruitment sites (black dots) for high dengue burden and circulation of 4 virus serotypes among children with undifferentiated fever, Kenya, 2014–2017. Inset map shows location of Kenya in Africa.

Children in Kenya who had undifferentiated febrile illness from 4 outpatient clinics located in Chulaimbo, Kisumu, Msambweni, and Ukunda were enrolled during January 24, 2014–August 15, 2017 (Figure 2). The clinics in Chulaimbo (a rural setting) and Kisumu (an urban setting) are located near Lake Victoria in western Kenya. Msambweni District Hospital (a rural setting) and Ukunda/Diani Health Centre (an urban setting) are located in coastal Kenya. All clinics are operated by the Kenyan Ministry of Health. Each child 1–17 years of age who came for care (Monday–Friday) because of an acute febrile illness (defined as reported illness during the previous 14 days and current observed temperature >38°C) and no localizing symptoms were recruited. Participants or their parents/guardians provided consent. We obtained detailed clinical histories and performed physical examinations. Comprehensive demographic and household data were recorded. Blood from each of the study participants was collected for serologic analysis, molecular testing, and malaria parasite smear. Data were stored in REDCap (*14*).

### RNA Extraction and cDNA Synthesis

An aliquot of whole blood from each participant was collected in Kenya. RNA was extracted by using the GeneJET RNA Purification Kit (ThermoFisher Scientific, https://www.thermofisher.com), and purified RNA was synthesized into complementary DNA (cDNA) by using the Maxima H Minus First Strand cDNA Synthesis Kit (ThermoFisher Scientific) according to the manufacturers’ instructions. All samples, including serum and cDNA, were shipped on dry ice and maintained at −80°C once received at Stanford University.

### RT-PCR and Envelope Gene Sequencing

PCR was performed at the Stanford Clinical Virology Laboratory. The rRT-PCRs used in this study, including a pan-DENV assay that detects all serotypes but does not distinguish between them, and a DENV serotyping assay, have been described (*15*,*16*). The pan-DENV assay uses primers and probes targeting RNaseP, which serves as an internal control. Specimens negative for DENV RNA with an internal control cycle threshold value >45 were considered to have failed extraction, cDNA generation, or PCR. Samples with cycle thresholds <35 by the pan-DENV assay or serotype-specific assay underwent sequencing attempts.

On the basis of the serotypes identified by rRT-PCR, serotype-specific envelope gene primers were used to generate amplicons for sequencing (Appendix Table 1). Each 25-μL reaction contained LongAmp Hot Start Taq 2× Master Mix (New England BioLabs, https://www.neb.com), 400 nmol/L of forward and reverse primers, 3% dimethyl sulfoxide, and 5 μL of cDNA. Amplification was performed in an Applied Biosystems Veriti 96-Well Thermal Cycler (ThermoFisher Scientific) by using the following cycling parameters: 94°C for 2 min; 40 cycles of 94°C for 30 s, 70°C for 1 s (ramp rate 20%), 55°C for 45 s (ramp rate 20%), and 65°C for 3 min, 20 s; and final extension for 10 min at 65°C. The PCR products were subjected to electrophoresis on a 1% agarose gel and visualized by staining with ethidium bromide. Dideoxynucleotide chain-termination sequencing (Sanger) was performed by Elim Biopharmaceuticals (https://www.elimbio.com) using the same primers used for amplification.

**Table 1:** Characteristics of participants by geographic location in study of dengue burden and circulation of 4 virus serotypes among children with undifferentiated fever, Kenya, 2014–2017*

Characteristic	Coastal clinics (Msambweni and Ukunda), n = 422	Western clinics (Chulaimbo and Kisumu), n = 600	p value
Mean (SD) age, y	6.7 (4.4)	4.6 (2.9)	<0.001
Sex			
F	188 (44.5)	279 (46.5)	0.58
M	234 (55.5)	321 (53.5)	0.58
Always uses bednet	400 (94.8)	418 (69.7)	<0.001
Wealth index <3†	272 (64.5)	329 (54.8)	0.003
Mean (SD) no. illness days before seeking care	2.3 (0.72)	3.1 (1.9)	<0.001
Dengue viremic by rRT-PCR	106 (34.2)	255 (46.2)	0.001
DENV IgG present at febrile visit	19 (4.5)	7 (1.2)	0.003
Malaria smear positive	194 (47.1)	247 (56.8)	0.006

*Values are no. (%) unless indicated otherwise. DENV, dengue virus; rRT-PCR, real-time reverse transcription PCR. †Defined as a household having <3 of the following: domestic worker, bicycle, telephone, radio, motor vehicle, and television.

### Whole-Genome Sequencing

Whole-genome sequencing was performed on a subset of convenience samples at the California Academy of Sciences’ Center for Comparative Genomics (https://www.calacademy.org). cDNA was purified by using the Zymo Clean and Concentrator Kit (Zymo Research, https://www.zymoresearch.com). Library preparation was performed by using the Nextera XT DNA Library Preparation Kit (96 samples) and the Nextera XT Index Kit (96 indices, 384 samples) (both from Illumina, https://www.illumina.com) according to manufacturer’s instructions with modifications: 3 ng of input cDNA with 4 μL index was used per reaction (rather than the suggested 1 ng input with 5 μL index because of low cDNA concentrations). Samples were tagged with a molecular identification tag. Libraries were quantified and assessed for quality on a BioAnalyzer (Agilent, https://www.agilent.com) before equimolar pooling for sequencing on the Illumina MiSeq platform using reagent run kits in series Nano, V2, and V3. Raw reads were uploaded by using CLC Genomics Workbench version 7.0.3 (https://www.qiagen.com) after being sorted by sample. DENV sample reads were mapped to complete, reference genome sequences downloaded from GenBank for each serotype. Virus consensus sequences were extracted in CLC Genomics Workbench version 7.0.3 and exported for phylogenetic analyses.

### Serologic Tests

Serum samples obtained at each patient visit were tested by using an indirect IgG ELISA for flavivirus IgG as (*17*). An ELISA result positive for DENV IgG in serum was indicative of previous exposure. Participants who had DENV IgG at the febrile visit were identified as having past exposure to dengue. All samples were tested in duplicate.

### Phylogenetic Analysis and Consensus Generation

Consensus sequences were deposited in GenBank under accession nos. MT076932–62. Multiple sequence alignment of both partial and complete genome sequences was performed by using the Multiple Alignment and Fast Fourier Transform plug-in in Geneious Prime 2020.0.5 (https://www.geneious.com). Reference genomes were selected to represent all genotype lineages and wide sampling of time periods focusing on sequences from Africa. To classify taxa, a maximum likelihood tree supported with maximum likelihood bootstrap replicates was inferred by using RAXML (https://raxml-ng.vital-it.ch) for each dengue serotype. Bootstrapping values were estimated starting with 100 replicates. The resulting trees were mid-point rooted to maintain consistency. Sylvatic strains were initially included to check for spillover, but then excluded from subsequent analyses to highlight differences in human-derived sequences.

### Statistical Testing and Cartography

Complete cases with cDNA specimens available for DENV rRT-PCR were included. Univariate analysis and a χ^2^ test for categorical variables and a *t*-test for continuous variables was used to compare participants on the basis of infection status with α = 0.05. One-way analysis of variance was used for >2 group mean comparisons. A comprehensive GenBank search term (Appendix Table 2) was used to obtain a list of original, verified, human dengue sequences from Africa through February 19, 2020. Maps were made by using QGIS version 3.4.14 (https://qgis.org).

**Table 2:** Clinical characteristics of febrile children stratified by dengue viremia status in study of dengue burden and circulation of 4 virus serotypes among children with undifferentiated fever, Kenya, 2014–2017*

Characteristic	Participants with dengue viremia, n = 361	Participants without dengue viremia, n = 661	p value
Mean (SD) age, y	5.2 (3.4)	5.6 (3.9)	0.13
Mean (SD) temperature, °C	38.8 (0.6)	38.9 (0.7)	0.14
Mean (SD) no. illness days before seeking care	2.8 (1.6)	2.7 (1.5)	0.21
Sex			
F	168 (46.5)	299 (45.2)	0.74
M	193 (53.5)	362 (54.8)	0.74
Signs and symptoms			
Headache	179 (49.6)	326 (49.3)	0.99
Poor appetite	169 (46.8)	240 (36.3)	0.001
Cough	165 (45.7)	278 (42.1)	0.29
Joint pain	133 (36.8)	259 (39.2)	0.50
Nausea or vomiting	128 (35.5)	219 (33.1)	0.50
Chills	66 (18.3)	118 (17.9)	0.93
Abdominal pain	46 (12.7)	134 (20.3)	0.003
Diarrhea	42 (11.6)	68 (10.3)	0.58
Muscle aches	30 (8.3)	54 (8.2)	1.00
Malaria smear positive	150 (51.5)	291 (52.3)	0.88
DENV IgG present at febrile visit	14 (4.0)	12 (1.9)	0.07
Prescribed antimicrobial drugs	182 (50.4)	290 (43.9)	0.05
Prescribed antimalarial drugs	176 (48.8)	243 (36.8)	<0.001

*Values are no. (%) unless indicated otherwise. DENV, dengue virus.

### Ethics

The study protocol was approved by the Stanford University Institutional Review Board (IRB-31488) and Kenya Medical Research Institute Scientific and Ethical Review Unit in Kenya (protocol 2611). Participation in the study did not affect routine clinical evaluation and treatment provided by the Ministry of Health.

## Results

During January 24, 2014–August 15, 2017, a total of 5,178 participants met eligibility for having an ongoing febrile illness without localization and documented body temperature >38°C when care was sought. Of eligible participants at clinics in the western region, 72.4% consented to participate in the study; 97.2% from clinics in the coastal region consented. RNA extraction and cDNA synthesis were successful for 1,022 specimens that were shipped to the United States for DENV nucleic acid analysis. The mean number of illness days before seeking care was 2 days (range 1–14 days). The study included 11 participants who had 2 separate visits for febrile illness. Of the 1,022 specimens tested by using the internally controlled, pan-DENV rRT-PCR, 15.7% (160/1,022) showed negative results for extraction, cDNA production, or PCR on the basis of the internal control and absence of DENV nucleic acid amplification. Dengue viremia was observed in 41.9% (361/862) of samples that passed quality control, including 46.2% (255/552) from western Kenya and 34.2% (106/310) from coastal Kenya. Overall, 92.2% (333/361) were classified as primary infections (DENV IgG not present) and 3.9% (14/361) as secondary infections (DENV IgG present); 3.9% (14/361) lacked DENV IgG data because of sample unavailability.

We provide baseline characteristics for participants by clinical site (Table 1). The mean age of enrolled children from the coastal region was higher than that for children from the western region (6.7 years vs. 4.6 years; p<0.001). Overall, girls were less represented than boys (467/1,022, 45.7%), and this trend was consistent across sites (44.5% girls in the coastal region, 46.5% girls in the western region). Bednet use was higher in the coastal region than in the western region (94.8% vs. 69.7%; p<0.001). Children in the coastal region had lower household wealth, defined as having <3 of a domestic worker, bicycle, telephone, radio, motor vehicle, or television (64.5% vs. 54.8%; p = 0.003). The number of illness days before seeking care was lower in the coastal region than in the western region (2.3 vs. 3.1 days; p<0.001). Malaria smear positivity was lower in the coastal region than in the western region (47.1% vs. 56.8%; p = 0.006) (Table 1). A higher proportion of febrile children in the western region had dengue viremia than children in the coastal region (46.2% vs. 34.2%; p = 0.001). Participants from the coastal region were more likely to be positive for DENV IgG at the febrile visit than participants from the western region (19/420 4.5% vs. 7/568 1.2%; p = 0.003) (Table 1). The mean age was 11.0 years for participants who were positive for DENV IgG compared with 5.4 years for participants who were negative for DENV IgG at the febrile visit (p<0.001).

Mean age of children with DENV detected by rRT-PCR did not differ from all others (5.2 years vs. 5.6 years; p = 0.13) (Table 2). In children with and without dengue, mean body temperature, sex, and malaria smear positivity were not different (Table 2). DENV viremic participants commonly reported headache (49.6%), poor appetite (46.8%), cough (45.7%), and joint pains (36.8%). Poor appetite was more often reported in dengue patients than in children without dengue (46.8% vs. 36.3%; p = 0.001). Antimicrobial drug prescriptions were significantly more common for children who had dengue viremia than for febrile patients without DENV (50.4% vs. 43.9%; p = 0.05). Antimalarial drug prescriptions were also more likely for DENV viremic patients (48.8% vs. 36.8%; p<0.001), although malaria smear positivity was not different between the groups (Table 2).

Among all 1,022 study participants, 419 (41.0%) were given an antimalarial. Malaria microscopy results were available for 847 (82.9%) participants; 441 (52.1%) were smear positive. Among study participants who had confirmed dengue and without malaria, 29/141 (20.6%) were given an antimalarial drug, 75/141 (53.2%) were given an antimicrobial drug, and 12/141 (8.5%) were given antimalarial and antimicrobial drugs. Fourteen (6.6%) of 213 participants who did not have dengue or malaria were given antimalarial and antimicrobial drugs.

**Figure 3:**
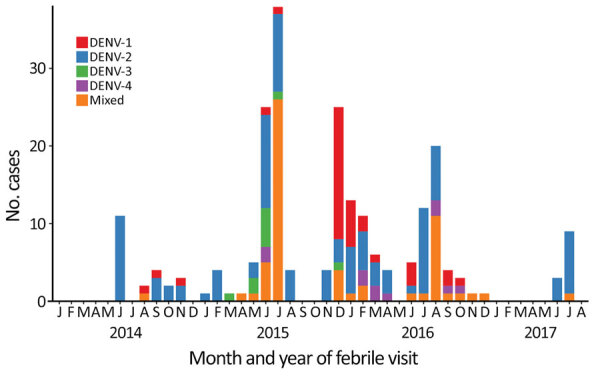
Dengue virus serotypes detected over time for children tested during a medical visit for fever, Kenya, 2014–2017. Data from 4 sites are included. DENV, dengue virus.

Serotypes were obtained for 61.2% (221/361) of DENV-positive samples. Samples that were able to be serotyped had higher mean virus loads than untypeable samples (6.5 log_10_ cDNA copies/µL vs. 4.6 log_10_ cDNA copies/µL; p<0.001). Of the serotyped samples, 16.3% (36/221) were DENV-1, 47.5% (105/221) were DENV-2, 4.5% (10/221) were DENV-3, 4.5% (10/221) were DENV-4, and in 26.7% (59/221) >2 serotypes were identified. Among the 59 mixed infections, the most common was DENV-2/DENV-3 (59.3%, 35/59); a total of 26/35 occurred during July 2015. The other serotype combinations were DENV-1/DENV-2 (20/59, 33.9%) and DENV-1/DENV-4 (4/59, 6.8%). Patients infected with DENV-3 had highest virus loads, but participant age, number of illness days before seeking care, and malaria smear positivity rate did not differ by serotype (Table 3). In Ukunda (urban coastal region), only DENV-2 was identified. In Msambweni, DENV-1 was the most common serotype. DENV-2 was most common serotype in Kisumu. DENV-4 was found mostly in Kisumu; there were a few cases in Msambweni. We provide the distribution of dengue serotypes over the study period (Figure 3).

**Table 3:** Characteristics of participants by DENV serotype in study of dengue burden and circulation of 4 virus serotypes among children with undifferentiated fever, Kenya, 2014–2017*

Characteristic	DENV-1, n = 36	DENV-2, n = 105	DENV-3, n = 10	DENV-4, n = 11	Mixed, n = 59	p value
Mean (SD) age, y	4.4 (2.8)	5.2 (3.3)	5.8 (3.9)	3.8 (1.6)	5.6 (3.3)	0.21
Mean (SD) viral load, log_10_ cDNA copies/μL	6.1 (2.5)	5.7 (2.2)	9.7 (3.8)	5.2 (2.7)	7.9 (1.8)	<0.001
Site						<0.001
Chulaimbo	5 (13.9)	53 (50.5)	2 (20.0)	1 (9.1)	26 (49.2)	
Kisumi	10 27.8)	28 (26.7)	7 (70.0)	10 (90.9)	25 (42.4)	
Msambweni	21 (58.3)	12 (11.4)	1 (10.0)	0	0	
Ukunda	0	12 (11.4)	0	0	0	
Mean (SD) no. illness days before seeking care	2.6 (1.6)	2.9 (1.9)	3.5 (1.5)	2.8 (1.47)	2.6 (1.3)	0.46
Malaria smear positive	18 (51.4)	42 (55.3)	3 (42.9)	4 (45.5)	23 (62.2)	0.78

*Values are no. (%) unless indicated otherwise. cDNA, complementary DNA; DENV, dengue virus.

**Figure 4:**
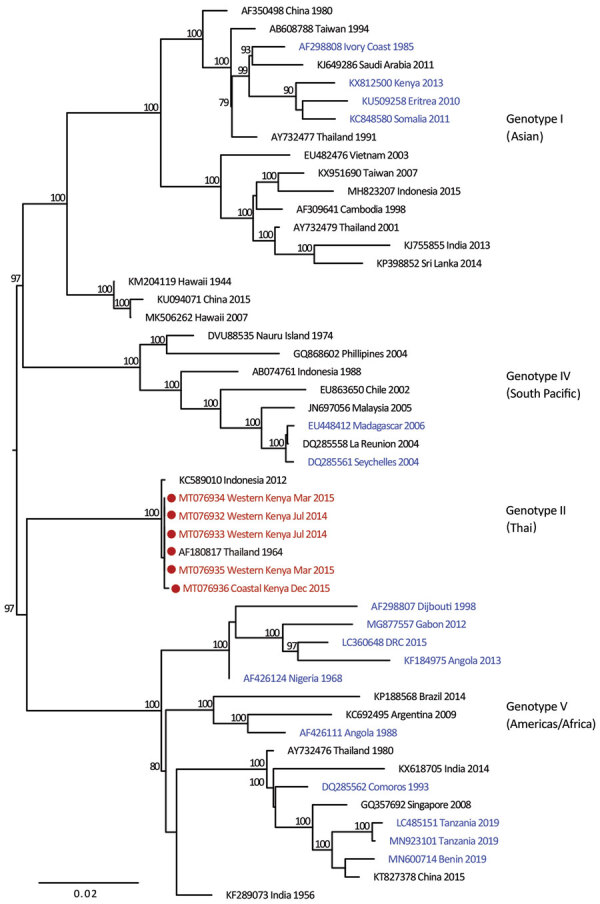
Maximum-likelihood phylogenetic tree for DENV-1 sequences from children with undifferentiated fever in Kenya, 2014–2017 (red), and reference sequences from other locations in Africa (blue) and elsewhere. Numbers at nodes represent bootstrap support values based on maximum-likelihood replicates. Genotypes are indicated at right. Scale bar indicates nucleotide substitutions per site. DENV, dengue virus.

**Figure 5:**
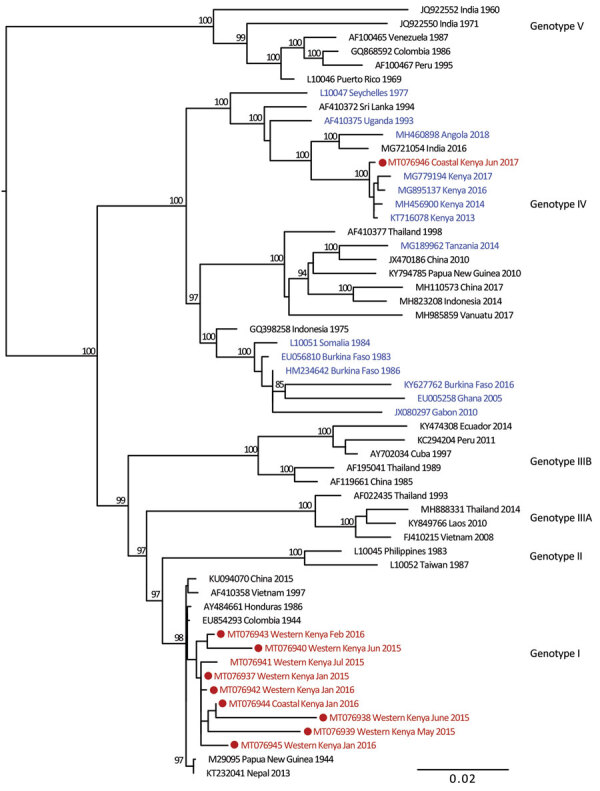
Maximum-likelihood phylogenetic tree for DENV-2 sequences from children with undifferentiated fever in Kenya, 2014–2017 (red), and reference sequences from other locations in Africa (blue) and elsewhere. Numbers at nodes represent bootstrap support values based on maximum-likelihood replicates. Genotypes are indicated at right. Scale bar indicates nucleotide substitutions per site. DENV, dengue virus.

**Figure 6:**
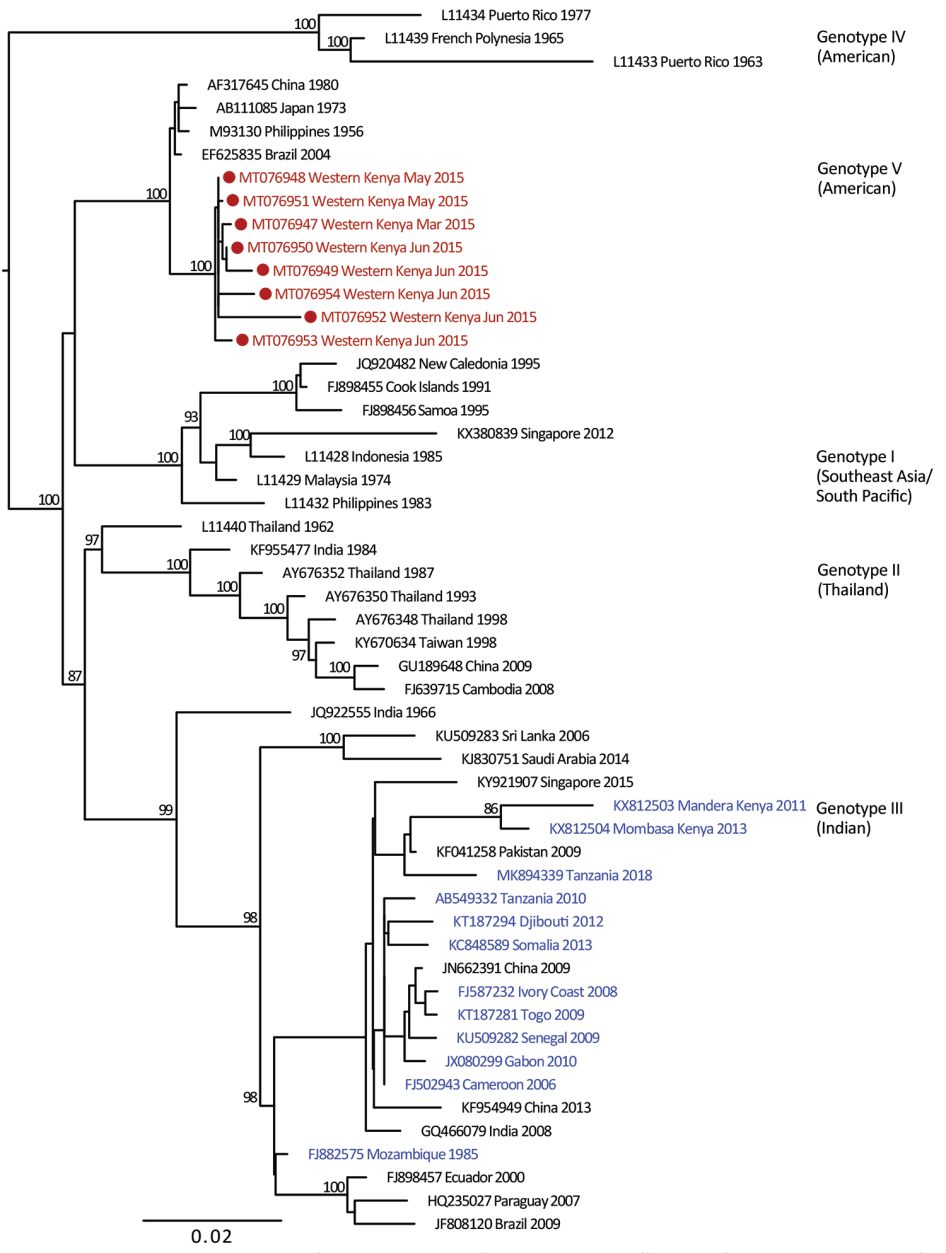
Maximum-likelihood phylogenetic tree for DENV-3 sequences from children with undifferentiated fever in Kenya, 2014–2017 (red), and reference sequences from other locations in Africa (blue) and elsewhere. Numbers at nodes represent bootstrap support values based on maximum-likelihood replicates. Genotypes are indicated at right. Scale bar indicates nucleotide substitutions per site. DENV, dengue virus.

**Figure 7:**
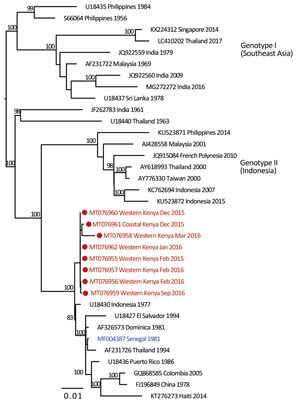
Maximum-likelihood phylogenetic tree for DENV-4 sequences from children with undifferentiated fever in Kenya, 2014–2017 (red), and reference sequences from other locations in Africa (blue) and elsewhere. Numbers at nodes represent bootstrap support values based on maximum-likelihood replicates. Genotypes are indicated at right. Scale bar indicates nucleotide substitutions per site. DENV, dengue virus.

We provide phylogenetic relationships of 31 DENV sequences, including 7 whole genomes, from the current study (Figures 4,5,6,7). The samples span July 2014–June 2017 and are derived from participants in coastal and western Kenya. Both DENV-2 and DENV-3 were sequenced from 1 participant sample (MT076939 and MT076951). Many of the samples belong to genotypes that differ from existing reference sequences from Africa. One DENV-2 sample in genotype IV aligns near reference sequences from Malindi, Kenya, and other countries in Africa, and all other DENV-2 samples cluster in genotype I (Figure 5).

## Discussion

Our study characterized the epidemiology of dengue among children with fever who came to clinics for outpatient care and the phylogenetic relationships of all 4 detected virus serotypes in Kenya. Of 1,022 febrile pediatric visits in western and coastal Kenya, >40% had evidence of dengue viremia. This unprecedented finding suggests an enormous burden of dengue fever among children with undifferentiated febrile illness in Kenya. The higher proportion of dengue in children from the western region than in children from the coastal region might be explained by a reported dengue outbreak in western Kenya during July 2014–June 2015 (*18*). Similar to previous findings showing 25% dengue viremia by RT-PCR for febrile patients in Kenya during 2011–2014 (*13*), the current study also identified a major proportion of febrile illness attributable to dengue. Similarly, Bhatt et al. estimated a burden of dengue disease in Africa similar to that in the Americas (>16% of the total global burden) (*19*). Our study supports the notion of an underrecognized dengue burden in Africa.

Our study identified circulation of multiple serotypes of DENV and adds valuable field strains to the public repository. Compilation of available DENV sequences from Africa into the phylogenetic analyses illustrates the dearth of sequences from Africa and is particularly evident for DENV-1 and DENV-4 (Figure 1). The DENV-1 samples align closely with a historical Thailand 1964 strain in genotype II. Although such a finding might suggest laboratory error or viral contamination, the Thailand 1964 strain is not a publicly available strain and has never been present in our laboratories. A DENV-1 strain in genotype II was curiously noted to cause human disease in Indonesia during 2012 and is similar to the Thailand 1964 strain (*20*). We postulate that viral strains related to the Thailand 1964 strain might be circulating at low levels in Kenya, but further surveillance for DENV-1 in other regions of Kenya and Africa is needed.

DENV-2 has been reported as the predominant serotype circulating in Africa (*2*). One study participant from coastal Kenya in June 2017 was infected with DENV-2 genotype IV, which clustered with previously reported DENV-2 sequences from Africa. However, 9 study samples from 2015 and 2016 clustered in genotype I and were related to sequences from China isolated during 2015 and Nepal during 2013. Commercial trade between Asia and Kenya has increased in recent years, and further sampling is needed to examine the role of such ties in the importation of dengue virus (*21*).

Several samples from western Kenya isolated during 2015 cluster in a DENV-3 lineage (genotype V, first documented in the Philippines in 1956). Samples from this lineage have been reported only rarely over the years, most recently in 2004 when they were recovered from human cases in Brazil (*22*). DENV-3 outbreaks and disease in returning travelers have been reported in Africa occurring within the genotype III (Indian) lineage, including the 2011 Mandera and 2013 coastal Kenya outbreaks (*13*,*23*). Observation of DENV-3 genotype V might indicate strains with low-level circulation endemically in eastern Africa or arrival by importation.

Of the 4 DENV serotypes, DENV-4 has been the least documented in Africa. During 2016, we documented a DENV-4 cluster in samples from western Kenya and in 1 sample from coastal Kenya. These serotypes group together in genotype II (Indonesia) in which related sequences come from the Americas and Asia. The recovery of DENV-4 sequences in our study was unexpected, in light of the paucity of DENV-4 documentation on the continent. Importation might be driver of dengue circulation in Kenya, but a clear source of DENV-4 introduction was not identified in this study. The findings suggest active DENV-4 circulation in eastern Africa for which robust surveillance is needed.

Human movement by modern transportation is a critical behavioral component underlying dengue circulation (*24*,*25*). The current study raises the possibility that dengue circulation in Africa might be driven by importations of disease, rather than by endemic strains. The similarity of some study samples to those of studies from Asia and Southeast Asia could also reflect movement along trade routes. Further sampling in Africa is required to unravel how the study isolates fit within the global circulation of DENV genotypes and to delineate the role of human movement in dengue transmission.

We document DENV circulation in Kenya over a 4-year period interspersed with small outbreaks as described (*17*). This pattern of disease is not consistent with large-scale dengue outbreaks flanked by long periods of disease quiescence as described in some settings (*26*). Whether underlying host susceptibility and host dengue viremia determine such patterns is an interesting avenue for further study, especially since persons of African ancestry might be less susceptible to dengue (*27*,*28*). Primary DENV infections comprised most of our samples, but interpretation is limited because our study was not representative of the general population.

Although there is no question that DENV circulates in Kenya, it is worth noting that the level of dengue we detected was surprisingly high. In addition, the number of concomitant serotype infections was also high and unexpected. The high percentage of dengue we document in febrile samples could be the result of the highly sensitive rRT-PCR used in this study (*16*). Another consideration is that RNA extraction was performed on whole blood as opposed to serum. In a study of hospitalized patients, when serum was compared with whole blood for rRT-PCR testing, whole blood yielded higher levels of dengue viremia and increased the detectable viremic window to 9 days (*29*). Testing of whole blood might have enabled detection of low-level viremia that would otherwise have not been detected in serum or plasma samples.

Half of the study participants who had dengue viremia also had malaria parasitemia. Some participants might have had fevers driven by malaria infection with asymptomatic dengue, and others had dengue plus asymptomatic malaria (*30*). This distinction is essential because asymptomatic dengue infections provide a major reservoir for human-to-mosquito dengue transmission (*19*). The higher rates of antimalarial and antimicrobial drug prescriptions for dengue viremic participants suggest more severe disease in this group and supports dengue as the driver of acute illness.

Our results confirm results of previous studies that showed overtreatment and diagnosis of malaria parasitemia (*31*,*32*). Persons with dengue symptoms should receive antibacterial and antimalarial therapies in the absence of point-of-care diagnostics for dengue. The similar clinical manifestations for persons with and without dengue also make treatment decisions difficult. Antimicrobial drug use for undifferentiated fever is a common practice, given challenges in ensuring close follow-up, expectations for prescriptions, and practice norms in developing countries. The consequences of untreated bacterial infections can be fatal because of limited access to care, but development of drug resistance and treatment for adverse effects must also be considered. Dedicated investments to develop a reliable dengue point-of-care diagnostic is urgently needed.

There are several limitations to this study. First, participants recruited were outpatients who had undifferentiated febrile illness and did not represent the general population. Studies designed to estimate dengue disease incidence and how febrile illness surveys can be extrapolated to estimate incidence are lacking. Sample testing by PCR occurred inconsistently throughout the study because of logistical considerations (availability of electricity, reliable transportation, access to laboratory equipment, strikes among healthcare workers), and the possibility of sampling bias exists. In addition, a proportion of total samples failed quality control measures because of suboptimal handling of specimens, inefficient nucleic acid extraction, or the presence of inhibitors in the PCR. This study cannot detangle the role of malaria versus dengue in symptomatic illness. Given the descriptive focus of this study, the clinical and statistical interpretation is limited, and further studies are needed to account for confounding and effect modification.

Our study provides insights into the phylogeny of DENV serotypes in eastern Africa and raises many more unanswered questions and avenues for further research. Phylogenetic analysis of circulating DENVs is crucial to clarifying movement of dengue into and throughout Africa. Viral movement will help identify dengue hotspots and ultimately provide clearer targets for prevention efforts. In addition, serotype circulation to the level of genotype is needed for assessing and predicting the efficacy of dengue vaccines. The paucity of dengue surveillance studies and sequence data from Africa is striking. Dengue activity will likely continue to spread in Africa because of rapid land use change, climate change, urbanization, increased human travel, and international trade (*33*). Knowledge of the spatial–temporal dynamics of dengue circulation throughout Africa is critically needed to inform a coordinated public health response in an increasingly interconnected world.

Appendix Additional information on high dengue burden and circulation of 4 virus serotypes among children with undifferentiated fever, Kenya, 2014–2017.
