# Supplementary material for: High Dengue Burden and Circulation of 4 Virus Serotypes among Children with Undifferentiated Fever, Kenya, 2014–2017
Source: Emerg Infect Dis. 2020 Nov;26(11):2638–50. doi: 10.3201/eid2611.200960 (PMC7588514; doi:10.3201/eid2611.200960)
Supplement: Appendix — Additional information on high dengue burden and circulation of 4 virus serotypes among children with undifferentiated fever, Kenya, 2014–2017. [file 20-0960-Techapp-s1.pdf]

Article DOI: https://doi.org/10.3201/eid2611.200960

High Dengue Burden and Circulation of 4 Virus Serotypes among Children with Undifferentiated Fever, Kenya, 2014–2017

Appendix

Appendix Table 1. Primers sequences for the dengue virus genotyping PCR*

Oligo name	Oligosequences, 5′→3′	Position	Length, bp	GenBank accession no.
D1_689F	GGTCTAGAAACAAGAACCGAAACATGGATG	704–733	1,547	MF004384.1
D1_2211R	ACACCGCTGAACAGAACCCCATAYG	2226–2250		
D2_750F2	ATGGATGTCATCAGAAGGGGCCTG	762–785	1,602	MG721057.1
D2_2300R2	ACAGACAGTGAGGTGCTACGTGAATT	2338–2363		
D3_750F1	GGGACTAGACACACGCACCCAAAC	739–762	1,682	MH544651.1
D3_2300R1	CCATGTCAGCTTGCACCACAGC	2399–2420		
D4_756F2	GATGTCATCGGAAGGGGCTTG	762–782	1,396	AY947539.1
D4_2137R2	TGCGCCTCTGTATGTGGACTC	2137–2157		

*Each pair is a forward and reverse primer. They have the same accession number and bp length because it is the start and end of an oligo. F, forward; R, reverse.

Appendix Table 2. GenBank search terms for human dengue virus sequences in Africa*

Search terms
Dengue virus 1 OR dengue virus type 1 OR dengue virus 2 OR dengue virus type 2 OR dengue virus 3 OR dengue virus type 3 OR dengue virus 4 OR dengue virus type 4 OR dengue virus isolate
AND (Algeria OR Angola OR Benin OR Botswana OR Burkina Faso OR Burundi OR Cabo Verde OR Cameroon OR Central African Republic OR Chad OR Comoros OR Democratic Republic of the Congo OR Cote d'Ivoire OR Djibouti OR Egypt OR Equatorial Guinea OR Eritrea OR Eswatini OR Swaziland OR Ethiopia OR Gabon OR Gambia OR Ghana OR Guinea OR Guinea-Bissau OR Kenya OR Lesotho OR Liberia OR Libya OR Madagascar OR Malawi OR Mali OR Mauritania OR Mauritius OR Morocco OR Mozambique OR Namibia OR Niger OR Nigeria OR Rwanda OR Sao Tome and Principe OR Senegal OR Seychelles OR Sierra Leone OR Somalia OR South Africa OR South Sudan OR Sudan OR Tanzania OR Togo OR Tunisia OR Uganda OR Zambia OR Zimbabwe) NOT Papua New Guinea

*This search strategy was used to develop an initial list that was further evaluated manually through February 10, 2020, to construct Figure 1.
